# Study Design, Rationale, and Baseline Characteristics: Evaluation of Fenofibric Acid on Carotid Intima-Media Thickness in Patients with Type IIb Dyslipidemia with Residual Risk in Addition to Atorvastatin Therapy (FIRST) Trial

**DOI:** 10.1007/s10557-012-6395-z

**Published:** 2012-05-24

**Authors:** Michael Davidson, Robert S. Rosenson, Kevin C. Maki, Stephen J. Nicholls, Christie M. Ballantyne, Carolyn Setze, Dawn M. Carlson, James Stolzenbach

**Affiliations:** 1 Radiant Research, 515 N. State St., Suite 2700, Chicago, IL 60610 USA; 2 Mount Sinai School of Medicine, New York, NY USA; 3 Provident Clinical Research, Glen Ellyn, IL USA; 4 Cleveland Clinic, Cleveland, OH USA; 5 Baylor College of Medicine, Houston, TX USA; 6 Methodist DeBakey Heart and Vascular Center, Houston, TX USA; 7 Abbott Laboratories, Abbott Park, IL USA

**Keywords:** Atorvastatin, CIMT, Dyslipidemia, Fenofibric acid, LDL particle, Triglycerides

## Abstract

**Purpose:**

Elevated triglycerides (TG) and low high-density lipoprotein cholesterol (HDL-C) levels contribute to cardiovascular disease risk and can be effectively treated with fenofibric acid. A trial is under way to evaluate the effect of once-daily fenofibric acid or placebo on carotid intima-media thickness (CIMT) progression in patients with controlled low-density lipoprotein cholesterol (LDL-C) levels achieved through atorvastatin treatment, but with high TG and low HDL-C levels.

**Methods:**

In this multicenter, double-blind study, 682 patients were randomized to once-daily delayed-release capsules of choline fenofibrate 135 mg (fenofibric acid [Trilipix®; Abbott, North Chicago, IL]) or placebo plus atorvastatin treatment after a 2- to 10-week diet and atorvastatin run-in period. Key inclusion criteria included age ≥45 years; posterior-wall common CIMT ≥0.7 mm on at least one side at baseline; fasting results of TG ≥150 mg/dL, and HDL-C ≤45 mg/dL for men or HDL-C ≤55 mg/dL for women at screening while receiving atorvastatin; controlled LDL-C; and known coronary heart disease (CHD) or a CHD risk equivalent. The primary efficacy variable is the rate of change from baseline through week 104 in the mean posterior-wall intima-media thickness of the common carotid arteries (composite value of left and right sides).

**Conclusions:**

This trial is the first to examine the effect of fenofibric acid on CIMT and the first CIMT trial to select patients with controlled LDL-C and elevated TG and low HDL-C as inclusion criteria. Also, this trial will prospectively evaluate the effect of treatment on LDL particles and address shortcomings of previous CIMT trials.

## Introduction

Herein we present the rationale and design of a trial with the objective of evaluating the effect of once-daily fenofibric acid or placebo, in addition to atorvastatin therapy, on carotid intima-media thickness (CIMT) progression in a population of patients with mixed (type IIb) dyslipidemia who have achieved low-density lipoprotein cholesterol (LDL-C) goals while receiving atorvastatin.

The association of elevated LDL-C with coronary heart disease (CHD) risk and the reduction of LDL-C with statin therapy are well documented. However, it is clear that the reduction of LDL-C by statin therapy alone does not always adequately reduce the risk of cardiovascular disease (CVD). In a review of several studies, Fruchart et al. reported the percentage of residual risk ranges from 63 % to 91 % [1], meaning that even when CVD events are reduced by reaching optimal LDL-C levels with statin therapy, many patients will still experience a CVD event. Factors contributing to residual risk include poor nutrition, lack of exercise, and dysregulation of lipids other than LDL-C (eg, high-density lipoprotein cholesterol [HDL-C] and triglycerides [TG]) [1].

Patients with mixed dyslipidemia (high LDL-C, low HDL-C, and high TG) may benefit from treatments that do more than just primarily lower LDL-C. One such treatment option is fenofibrate, or its active metabolite, fenofibric acid, which increases HDL-C and lowers TG. In a meta-analysis of 18 randomized controlled trials, fibrate monotherapy was found to significantly reduce major cardiovascular (CV) events, coronary events, and non-fatal coronary events compared with placebo [2]. For fenofibrate specifically, results from the ACCORD study determined that of patients treated only with simvastatin, the CVD event rate in the subset of patients with mixed dyslipidemia (TG ≥204 mg/dL and HDL-C ≤34 mg/dL) was quite high compared with the remainder of the patient population (17.3 % vs 10.1 %, respectively); add-on fenofibrate therapy resulted in a lower CVD event rate of 12.4 % in this mixed dyslipidemia subset [3]. In addition, in a post hoc analysis of the FIELD study, an apparent benefit of fenofibrate monotherapy treatment on CVD risk, relative to placebo, in patients with type 2 diabetes treated for a median of 5 years was observed in those with baseline TG ≥204 mg/dL and HDL-C <40 mg/dL for men and <50 mg/dL for women [4]. These possible benefits of fenofibrate therapy in the ACCORD and FIELD studies were only observed in the mixed dyslipidemia subgroups, whereas no significant reductions in CVD event rates were observed in the overall study populations [3, 5].

Another meta-analysis that included a total of 4,726 patients from the ACCORD and FIELD studies, as well as 3 studies on the fibrates gemfibrozil and bezafibrate, specifically evaluated treatment effect on coronary outcomes in the subsets of patients with mixed dyslipidemia, defined as TG ≥204 mg/dL and HDL-C ≤34 mg/dL [6]. The odds of experiencing a CHD event were reduced with fibrate treatment by 35 % compared with placebo in the dyslipidemia cohort, and only 6 % in a matched cohort without dyslipidemia. These data highlight the potential benefit of fibrate treatment for patients with mixed dyslipidemia, but until now, no clinical study has been designed to specifically evaluate the effect of fibrates in this population.

Warnings concerning a possible increased risk for muscle-related adverse events associated with a combination of fibrate and statin have limited its use; however, long-term studies with a choline salt of fenofibric acid or fenofibrate and statin therapy have demonstrated that this combination is generally well tolerated [3, 5, 7–11]. The incidence of muscle-related adverse events was similar between combination treatment and statin monotherapy [9]. Thus, the Food and Drug Administration has approved an indication for the choline salt of fenofibric acid formulated as delayed-release mini-tablets in a capsule (fenofibric acid [Trilipix®]; Abbott, North Chicago, IL) for use in combination with a statin to reduce TG and increase HDL-C in patients with mixed dyslipidemia and CHD or a CHD risk equivalent who are on optimal statin therapy to achieve their LDL-C goal [12]. Several 12-week controlled studies of combination fenofibric acid plus statin therapy have demonstrated significant benefits on HDL-C, TG, non-HDL-C, apolipoprotein (Apo) B, and very low-density lipoprotein cholesterol (VLDL-C) levels compared with statin treatment alone [13–16]. These benefits were maintained for at least 2 years [17] and were also demonstrated in subpopulations of patients with diabetes mellitus or persistently elevated TG [18, 19]. Safety profiles were similar between combination treatments and monotherapies, with no unexpected safety signals relative to each of the monotherapies.

Carotid intima-media thickness is a measure of subclinical atherosclerosis. The association between CVD and CIMT is well established, with abundant evidence that increased CIMT is a risk factor for CV events [20–23]. Predictors of CIMT progression have included elevated TG, non-HDL-C, Apo B, and various lipid ratios in patients with moderate CHD risk [24]. CIMT can also be used as a tool for CVD risk assessment [25–27]. Many CVD drug studies have utilized CIMT as a clinical endpoint, although there are differing opinions on the validity of CIMT as a surrogate marker for outcome studies [28, 29]. A unique aspect of the current study is that it is the first to include only patients with high TG, low HDL-C, and controlled LDL-C. The selection of this patient population will enable further elucidation of the relationship between the pharmacologic modifications of TG and HDL-C, and the progression of atherosclerosis.

A previous study investigated the effect of fenofibrate plus antihypertensive treatment on CIMT in 225 patients with essential hypertension and high TG [30]. After 2 years of combination treatment, CIMT measures either remained stable or regressed. In contrast, patients on antihypertensive therapy alone had significant progression of CIMT measures. Also of note, treatment with fenofibrate alone in a study population with type 2 diabetes and normal TG and HDL-C did not affect CIMT, but did significantly reduce TG compared with baseline (*P* < 0.001) [31].

An abundance of small LDL particles is common in patients with elevated TG and low HDL-C. These small particles may be particularly atherogenic because of their greater propensity to enter the arterial wall, where they can undergo oxidation and stimulate endothelial cell production of inflammatory proteins and procoagulants [32]. Uncertainty remains as to whether LDL size is independently associated with CVD risk after adjusting for the LDL particle concentration. The current study will prospectively evaluate the effect of fenofibric acid treatment on LDL particle size and concentration, providing an opportunity to assess the relationships between LDL particle concentration and size, as well as changes in these parameters and risk for CIMT progression.

The current trial was designed to address some of the limitations of previous CIMT trials. For example, patients selected for the trial should be at high risk for CIMT progression and have sufficient CIMT thickness to allow potential for CIMT regression. The lack of a minimum CIMT thickness entry criterion has been suggested as a possible reason patients in the ENHANCE trial did not exhibit significant improvement in CIMT in response to ezetimibe plus simvastatin treatment compared with simvastatin alone [33]. In contrast, other trials with minimum CIMT requirements have demonstrated significant treatment-specific CIMT reductions [34, 35]. The current trial only includes patients with an increased CIMT (common carotid ≥0.7 mm) at baseline, which may increase the probability of detecting a treatment effect.

In the current study, particular consideration was given to the assumptions included in the power analysis. Thirteen studies that used simple mean change from baseline (7 studies) or regression models (6 studies) were analyzed to determine the common standard deviation (SD) for the annual progression rate (Table 1) [31, 34–46]. Regression-based models would generally be expected to result in smaller SDs because they reduce the unexplained variance due to the correlation of the repeated measures within each patient. The median SD from the studies using regression models was substantially lower (0.028) than that for the studies analyzing the simple mean change from baseline (0.076). Thus, the assumption of SD = 0.045 in this study is expected to be conservative due to the use of the mixed-model regression analysis.

**Table 1:** Comparison of baseline lipid levels and mean common CIMT progression rates from clinical studies used to calculate the power analysis standard deviation

Study Treatment	Mean BaselineLDL-C (mg/dL)^a^	Mean BaselineHDL-C (mg/dL)^a^	Mean Baseline TG (mg/dL)^a^	Treatment Period Duration	Mean Common CIMT Change from Baseline, mm (SD)
Taylor et al. (2004) [37]					
Niacin	87	39	154	12 months	0.014 (0.104)
Placebo	91	40	172		0.044 (0.100)
Difference					−0.03
Sidhu et al. (2004) [44]					
Rosiglitazone	98	45	114	48 weeks	−0.012 (0.094)
Placebo	102	47	137		0.031 (0.096)
Difference					−0.043
Hanefeld et al. (2004) [46]					
Acarbose	Not	51	212	≥36 months	0.007 (0.019)^b^
Placebo	reported	49	235		0.013 (0.018)^b^
Difference					−0.006^b^
Mazzone et al. (2006) [36]					
Pioglitazone	113.8	47.1	178.6	72 weeks	−0.001
Glimepiride	111.3	47.6	170.4		0.012
Difference					−0.013
Hodis et al. (2006) [42]					
Troglitazone	170	50	115	24 months	0.0030 (0.021)^b^
Placebo	182	50	115		0.0066 (0.021)^b^
Difference					−0.0036^b^
Hedblad et al. (2007) [41]					
Rosiglitazone	135	50	150	12 months	0.01 (0.073)
Placebo	131	50	150		0.017 (0.076)
Difference					−0.007
Crouse et al. (2007) [34]					
Rosuvastatin	155	50	126	24 months	0.0004 (0.019)^b^
Placebo	154	49	134		0.0088 (0.02)^b^
Difference					−0.0085^b^
Kastelein et al. (2007) [38]					
Atorvastatin	139	52	97^c^	24 months	−0.0014 (0.027)^b^
Atorvastatin + torcetrapib	138	53	97^c^		0.0038 (0.027)^b^
Difference					−0.0052^b^
Kastelein et al. (2008) [43]					
Simvastatin	318	47	160^c^	24 months	0.0024 (0.077)
Simvastatin + ezetimibe	319	47	157^c^		0.0019 (0.079)
Difference					0.0005
Hiukka et al. (2008) [31]					
Fenofibrate	120	45	136	60 months	0.0050 (0.060)^b^
Placebo	118	43	147		0.0069 (0.054)^b^
Difference					−0.0019^b^
Meuwese et al. (2009) [35]					
Pactimibe	141	51	135	12 months	0.019 (0.099)^d^
Placebo	139	52	136		0.005 (0.085)^d^
Difference					0.014
Taylor et al. (2009) [45]					
Ezetimibe	84	43	122^c^	14 months	−0.0007 (0.037)
Niacin	81	43	126^c^		−0.0142 (0.040)
Difference					0.0135
Davidson et al. (2009) [40]					
Pomegranate juice	139	55	153	18 months	0.005 (0.048)^b^
Control	142	56	144		0.005 (0.048)^b^
Difference					0.00^b^

*C* cholesterol; *CIMT* carotid intima-media thickness; *HDL* high-density lipoprotein; *LDL* low-density lipoprotein; *TG* triglyceride

^a^Reported values in mmol/L were converted to mg/dL by dividing cholesterol values by 0.02586 and TG values by 0.01130

^b^Change per year

^c^Median values

^d^Included common, bulb, and internal segments

The hypothesis for the current study is that patients with mixed dyslipidemia treated with a statin to reach prespecified LDL-C levels, but with persistent high TG and low HDL-C levels, will experience CIMT benefits from the lipid-altering effects of fenofibric acid.

## Methods

### Study design

**Fig. 1:**
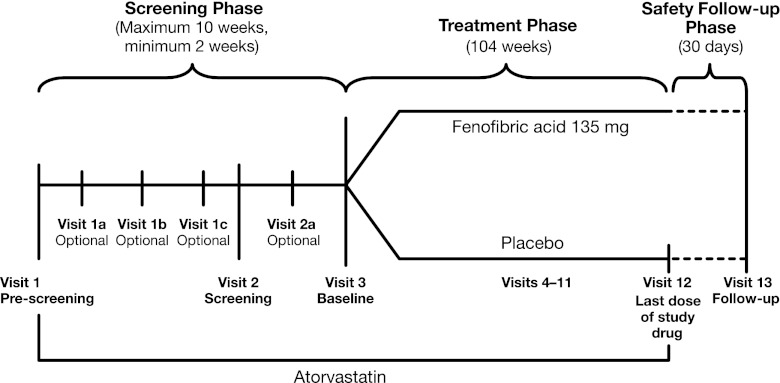
Study design

This is a randomized, multicenter, prospective, double-blind, placebo-controlled, phase III study of 682 patients in the United States (80 sites in 12 geographic locations; ClinicalTrials.gov identifier: NCT00616772). The planned duration of the study is 118 weeks, composed of a 2- to 10-week diet and atorvastatin run-in period, a 104-week treatment period, and a 30-day safety follow-up period (Fig. 1). The estimated study completion date is August 2012. Written informed consent was obtained from all patients, and institutional review board approval of the protocol and informed consent form was obtained from each study site.

### Key inclusion/exclusion criteria

Patients meeting the following criteria were included: age ≥45 years at visit 1; posterior-wall intima-media thickness (IMT) of the common carotid artery (CCA) ≥0.7 mm on one side at baseline; TG ≥150 mg/dL and HDL-C ≤45 mg/dL for men or HDL-C ≤55 mg/dL for women after a ≥12-hour fasting period at screening; LDL-C ≤100 mg/dL at visit 1, 1a, 1b, or 1c AND an average of 2 consecutive LDL-C values ≤105 mg/dL from visits 1, 1a, 1b, 1c, and visit 2. Patients were also required to have known CHD or a CHD risk equivalent. Patients with any of the following were excluded from the study: systolic blood pressure >140 mm Hg or diastolic blood pressure >90 mm Hg at screening, type 1 diabetes mellitus, diabetic ketoacidosis, or uncontrolled type 2 diabetes mellitus (HbA1c of >10.5 %).

### Randomization procedure

A randomization schedule stratified by baseline atorvastatin dose assigned patient numbers to blinded treatment assignments (fenofibric acid or placebo) in a 1:1 ratio. An additional randomization schedule assigned study drug bottle numbers to blinded treatment assignments, and a random subset of study drug bottles were then supplied to each site. At visit 1, the patient was assigned a unique screening number through the use of an Interactive Voice Response System (IVRS). At baseline, the assigned screening number and the current atorvastatin dose was given to the IVRS by the site. The IVRS then assigned a 4-digit patient number and one of the study drug bottle numbers corresponding to the randomized treatment assignment to each patient.

### Study phases

#### Screening phase

At visit 1 (prescreening), all patients had a fasting blood sample drawn for blood chemistry, HDL-C, TG, and LDL-C levels. Patients currently taking atorvastatin and with a LDL-C level of ≤100 mg/dL (goal LDL-C) proceeded to visit 2 (screening) within 1 to 2 weeks. Patients currently taking atorvastatin, but with an LDL-C level of >100 mg/dL, had their atorvastatin dose titrated to reach their LDL-C goals. Up to 3 visits (visit 1a, b, and c) separated by approximately 2 weeks were allowed to achieve the LDL-C goal before proceeding to visit 2 (within 1–2 weeks of achieving the LDL-C goal). Patients not currently taking atorvastatin discontinued other statins and began treatment with atorvastatin, with up to 3 visits (visit 1a, b, and c) approximately 2 weeks apart allowed for dose titration to achieve their LDL-C goal before visit 2. All patients must have been taking atorvastatin for at least 4 weeks before visit 3 (baseline). During the screening phase, all patients also initiated a diet recommended by the American Heart Association [47].

#### Treatment phase

At baseline, patients were randomly assigned to either a once-daily delayed-release capsule of choline fenofibrate 135 mg (fenofibric acid [Trilipix®]) or once-daily placebo in addition to atorvastatin treatment. To maintain an LDL-C value of <130 mg/dL, investigators reinforced diet requirements and adjusted the atorvastatin dose (maximum dose 40 mg/d). The addition of ezetimibe (10 mg) was allowed if the patient was already at the maximum atorvastatin dose. To ensure all personnel and patients remained blinded following the baseline visit, a central laboratory reviewed LDL-C values and informed the investigator if the atorvastatin treatment required modification. Additional visits were scheduled for weeks 6, 13, 26, 39, 52, 65, 78, 91, and 104.

#### Safety follow-up phase

The follow-up phase will begin 1 day after the last dose of study drug and end approximately 30 days later.

### Procedures and assessments

#### Physical examination and laboratory analysis

A complete physical examination was conducted at baseline and a symptom-directed (per patient report or examiner identified) physical was performed at all subsequent visits. Vital signs, including sitting blood pressure, heart rate, body temperature, and weight, were measured at all visits. Blood and urine were collected at all visits (except no urinalysis at visit 1), with a fasting period of at least 12 h before the blood draw. The lipid and inflammatory parameters to be assessed are listed in Table 2. A 12-lead resting electrocardiogram (ECG) assessment was performed at baseline and the final visit.

**Table 2:** Lipid and inflammatory markers to be assessed

Lipid Markers	Inflammatory Markers	Lipid Particle Sizes and Concentrations by NMR Spectroscopy
Total-C	hs-CRP	VLDL (total, large, medium and small)
LDL-C (direct)		LDL (total, IDL, large and small)
HDL-C		HDL (total, large, medium and small)
TG		
TG:HDL ratio		
Non-HDL-C		
VLDL-C		
Apo AI		
Apo AII		
Apo B		
Apo B:Apo AII ratio		
Apo CIII associated with Apo B-containing lipoprotein particles		

*Apo* apolipoprotein; *C* cholesterol; *HDL* high-density lipoprotein; *hs-CRP* high-sensitivity C-reactive protein; *IDL* intermediate-density lipoprotein; *LDL* low-density lipoprotein; *NMR* nuclear magnetic resonance; *TG* triglyceride; *VLDL* very low-density lipoprotein

#### Carotid intima-media thickness

**Fig. 2:**
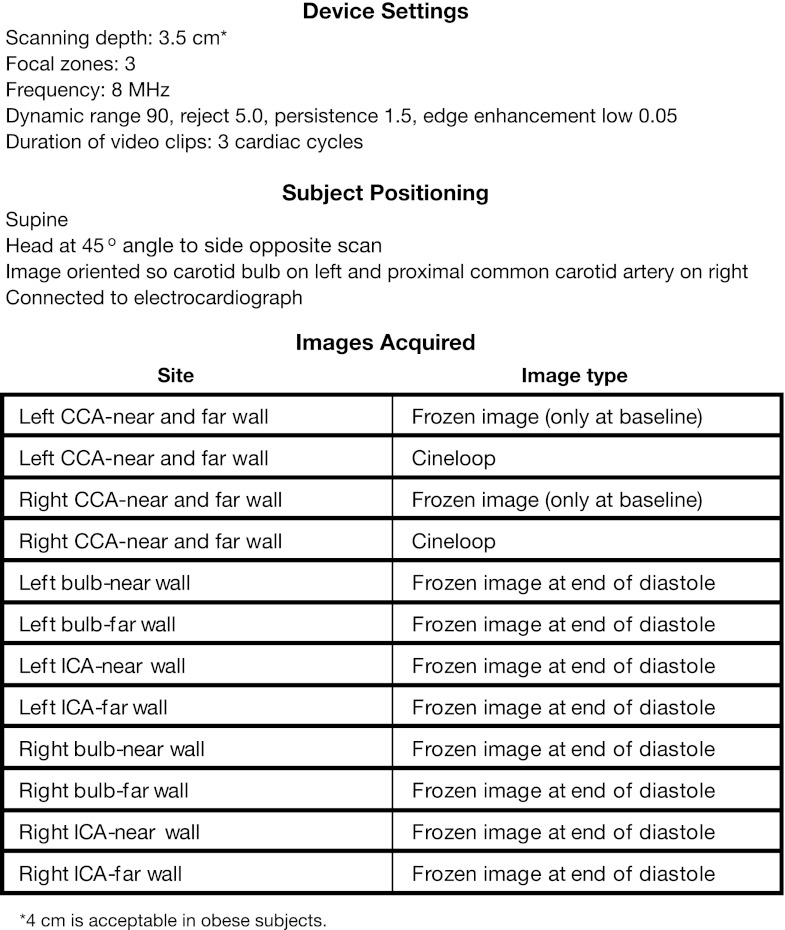
Scanning protocol CCA = common carotid artery; ICA = internal carotid artery

Carotid intima-media thickness was measured within 17 days before baseline and at weeks 26, 52, 78, and 104 (or upon premature discontinuation). CIMT was only measured upon premature discontinuation if a patient received a minimum of 26 weeks of study drug treatment. The procedure was done by a trained sonographer at a central facility within each of the geographic locations of the participating sites using a standardized protocol (Fig. 2). All facilities used the same device (GE VIVID i equipped with an 8-L probe). A mask tool was used to allow proper repositioning during follow-up visits. After the sonographer selected the region of interest, the CIMT detection and measurement was fully automated.

#### Safety

Safety evaluations included physical examinations, vital signs, clinical laboratory testing (hematology, chemistry, and urinalysis), ECG measurements, and adverse event monitoring. Each adverse event was rated by severity and relationship to the study drug.

#### Compliance

Compliance with dietary instructions was assessed at all visits following visit 1 based on patient self-reporting. Each patient was asked to return the previously dispensed study drug bottle for documentation of study drug compliance. Patients were considered to be compliant if they adhered to ≥80 % of dosing.

#### Endpoints

The primary efficacy variable is the rate of change from baseline through week 104 of the mean posterior-wall IMT of the left and right CCAs. The secondary efficacy variables are the rates of change from baseline through week 104 in mean of the maximal posterior-wall IMT of the left and right CCAs and the composite of the CCAs, internal carotid, and carotid bifurcation. Additional efficacy variables include:

1. Rate of change from baseline to week 104 in:
  - Mean of the median, 10th percentile, and 90th percentile posterior-wall IMT of the left and right CCAs
  - Mean cross-sectional area (calculated using posterior-wall IMT and diameter) of the left and right CCAs
2. The occurrence of CV events as a composite of CV mortality, nonfatal myocardial infarction (MI), and nonfatal stroke
3. The occurrence of CV events as a composite of CV mortality, nonfatal MI, nonfatal stroke, coronary revascularization, carotid endarterectomy/stenting, hospitalization for unstable angina, and hospitalization for congestive heart failure
4. Change from baseline in lipid profile and inflammatory markers listed in Table 2

### Statistical considerations

#### Power analysis

A sample size of 300 to 350 patients per group provides 92 % to 95 % power to detect a 0.014 mm/y change in CIMT, with a 2-sided α-level of 0.05, assuming a common standard deviation of 0.045 mm/y and allowing for a 25 % dropout rate. The power and sample size were revised from the initial protocol (originally 400 patients per group) based on 1) a reassessment of the literature, resulting in a lower standard deviation for CIMT progression (originally 0.050), and 2) a lower than expected discontinuation rate, resulting in a larger evaluable sample than originally projected.

#### Outcome analysis

Means of demographic characteristics will be compared between treatment groups using a one-way analysis of variance (ANOVA). The frequencies and percentages for demographic characteristics will be compared between treatment groups using a chi-square test. Baseline values for CIMT and efficacy laboratory parameters will be compared between treatment groups using a two-way ANOVA with effects for baseline atorvastatin dose and treatment group. Primary, secondary, and additional CIMT variables assessing rates of change will be analyzed using a repeated measures linear mixed-effects model with fixed effects for baseline mean CIMT value, site, baseline atorvastatin dose, treatment group, time, and the interaction between treatment group and time. Time to first CV events will be compared between treatment groups using the log-rank test stratified by baseline atorvastatin dose. A Cox proportional hazards model with baseline atorvastatin dose as a covariate will be used to obtain the hazard ratio and 95 % confidence interval for the hazard ratio. The percentage changes from baseline in all efficacy laboratory parameters (except high-sensitivity C-reactive protein [hs-CRP]) will be analyzed using an analysis of covariance with baseline values (laboratory parameter corresponding to the efficacy variable being modeled) as the covariate and baseline atorvastatin dose and treatment group as effects. A non-parametric analysis will be performed to evaluate the percentage changes from baseline in hs-CRP. Mean changes from baseline in safety laboratory parameters will be compared between treatment groups using a one-way ANOVA. *P* values ≤0.05 will be considered statistically significant.

## Results

The demographics and baseline characteristics of the study population are shown for the treated patients in Table 3 and clinical characteristics are shown in Table 4. The population is not broken down by treatment group since the study is ongoing, and hence blinded. The characteristics are in alignment with the targeted patient population, although the Apo B level is surprisingly low in relation to LDL-C levels and considering non-HDL-C levels. However, previous CIMT studies may have used different measurements of LDL-C. It is unclear how this parameter will affect the outcome and interpretation of the study.

**Table 3:** Demographics and baseline characteristics of the total blinded treated patient population

Variable	Total Patients (*n* = 676)
Male, n (%)	458 (67.8)
White race, n (%)	593 (87.7)
Age	
Mean, y (SD)	60.8 (8.75)
Range, y	45–87
Mean BMI, kg/m^2^ (SD)	32.7 (5.81)
Nicotine use, n (%)^a^	
User	153 (22.7)
Ex-user	272 (40.3)
Non-user	250 (37.0)
Mean lipid parameters, mg/dL (SD)	
Total-C	157.9 (26.2)
LDL-C (direct)	84.3 (20.9)
HDL-C	39.8 (7.6)
TG	227.5 (118.8)
Non-HDL-C	118.0 (24.9)
VLDL-C	41.7 (20.4)
Apo B	81.0 (15.6)
Apo AI	130.7 (17.9)
Apo AII	34.8 (5.7)
Apo CIII-lipoprotein B^b^	1.6 (0.8)
hs-CRP, mg/L (SD)	4.1 (7.8)
Mean HDL particles, μmol/L (SD)	
Total	32.3 (4.9)
Small	25.1 (5.3)
Medium	3.3 (3.8)
Large	3.9 (1.9)
Mean LDL particles, nmol/L (SD)	
Total	1,118 (277)
Small	919 (275)
Large	153 (112)
IDL	46 (38)
Mean VLDL particles, nmol/L (SD)	
Total	93 (35)
Small	38 (17)
Medium	48 (23)
Large	8 (7)
VLDL TG, mg/dL (SD)	155.4 (83.2)

*Apo* apolipoprotein; *BMI* body mass index; *C* cholesterol; *HDL* high-density lipoprotein; *hs-CRP* high-sensitivity C-reactive protein; *IDL* intermediate-density lipoprotein; *LDL* low-density lipoprotein; *TG* triglyceride; *VLDL* very low-density lipoprotein

^a^Includes all types of nicotine use

^b^Apo CIII associated with lipoproteins that contain Apo B, excluding Apo CIII associated with HDL particles

**Table 4:** Clinical characteristics of the total blinded treated patient population

Condition, *n* (%)	Total Patients (*n* = 676)
Angina	63 (9.3)
Cardiac arrhythmia	55 (8.1)
Carotid artery disease	44 (6.5)
Congestive heart failure	13 (1.9)
Coronary artery disease	146 (21.6)
Diabetes type 2	337 (49.9)
Emphysema/COPD	44 (6.5)
Hypertension	532 (78.7)
Myocardial infarction	72 (10.7)
Obesity	193 (28.6)
Peripheral vascular disease, arterial	16 (2.4)
Peripheral vascular disease, venous	5 (0.7)
Transient ischemic attack	13 (1.9)
Valvular heart disease	16 (2.4)

*COPD* chronic obstructive pulmonary disease

## Summary

In summary, the current study is novel in several ways:

- It is the first study to examine the effect of fenofibric acid on CIMT.
- It is the first CIMT trial to select patients with controlled LDL-C and elevated TG and low HDL-C as inclusion criteria.
- Prospective analysis of LDL particle size and concentrations, as well as Apo B and Apo CIII on Apo B-containing lipoprotein particles, may provide more information on the association of these parameters with risk for CIMT progression compared with LDL-C.
- The trial was designed to address some of the limitations of previous CIMT trials.

A beneficial outcome on CIMT after treatment would provide evidence that fenofibric acid can not only improve lipid parameters, but also have a positive effect on progression of subclinical atherosclerosis.
